# Speaking and Listening with the Eyes: Gaze Signaling during Dyadic Interactions

**DOI:** 10.1371/journal.pone.0136905

**Published:** 2015-08-26

**Authors:** Simon Ho, Tom Foulsham, Alan Kingstone

**Affiliations:** 1 Department of Psychology, University of British Columbia, Vancouver, British Columbia, Canada; 2 Department of Psychology, University of Essex, Colchester, Essex, United Kingdom; University of Lincoln, UNITED KINGDOM

## Abstract

Cognitive scientists have long been interested in the role that eye gaze plays in social interactions. Previous research suggests that gaze acts as a signaling mechanism and can be used to control turn-taking behaviour. However, early research on this topic employed methods of analysis that aggregated gaze information across an entire trial (or trials), which masks any temporal dynamics that may exist in social interactions. More recently, attempts have been made to understand the temporal characteristics of social gaze but little research has been conducted in a natural setting with two interacting participants. The present study combines a temporally sensitive analysis technique with modern eye tracking technology to 1) validate the overall results from earlier aggregated analyses and 2) provide insight into the specific moment-to-moment temporal characteristics of turn-taking behaviour in a natural setting. Dyads played two social guessing games (*20 Questions* and *Heads Up*) while their eyes were tracked. Our general results are in line with past aggregated data, and using cross-correlational analysis on the specific gaze and speech signals of both participants we found that 1) speakers end their turn with direct gaze at the listener and 2) the listener in turn begins to speak with averted gaze. Convergent with theoretical models of social interaction, our data suggest that eye gaze can be used to signal both the end and the beginning of a speaking turn during a social interaction. The present study offers insight into the temporal dynamics of live dyadic interactions and also provides a new method of analysis for eye gaze data when temporal relationships are of interest.

## Introduction

When interacting with another person, we coordinate our behaviour in order to take turns in the conversation. Dyadic interactions, and turn-taking behaviour in particular, has received considerable attention in cognitive science, typified by several models of social interaction (e.g., [1,2]). These models have since been tested and quantified using both aggregated and temporally sensitive techniques. Cognitive scientists are beginning to include realistic interactions when investigating social attention in recognition of the fact that these introduce critical factors that are excluded during simulated social settings [3–6]. Accordingly, the current study investigates the role of social gaze in interacting dyads, using modern eye tracking techniques and analyses, to determine the temporal dynamics inherent to natural interactions.

### Natural interactions

Research on attention has typically been conducted using simple, laboratory paradigms and these studies have been pivotal to our understanding of how we select information both with and without overt shifts of gaze. However, there is increasing evidence that findings from laboratory paradigms do not always translate to natural, real world situations. While we are not studying gaze cueing in the present study, research in that domain highlights this difference very clearly. For example, an attentional orienting effect has been found whereby people are faster to respond to stimuli at cued versus non-cued locations [7], and this effect also occurs when the cueing stimulus is socially relevant (e.g., a human face looking in a particular direction [8,9]). Real world studies of attention have shown, however, that gaze cueing can operate differently when other people are present. Gallup, Chong and Couzin [10] demonstrated that people are less likely to follow the gaze of another person if that person can see them.

Beyond cueing, gaze behaviour in general is found to be different in natural situations. For example, participants often gaze at people in video, but participants gaze less frequently and for a shorter duration at the same people when these individuals are physically present in a real-life situation [11,12]. The potential for interaction in a real life encounter seems to change gaze behaviour relative to artificial lab based studies (for an extensive review of the differences between lab and real world gaze behaviour, see Risko et al. [3]).

Given that social attention is often exhibited differently in real-life situations, researchers wishing to understand how it operates in everyday life will need to examine it in its natural settings. If the goal is to study attention in a social context, then describing how gaze functions in natural, dyadic interactions is a good place to start, not least because of the past progress made in describing this general phenomenon, as we report below. Specifically, in the following sections we will describe some of the behavioural patterns that have been found during face-to-face interactions.

### Gaze and aggregated methods

Natural social dyadic interactions are segmented into turns, and there appear to be some cross-cultural universals in how turn-taking operates [13]. Turn-taking behaviour is ubiquitous, and is understood to occur as a result of social signals (verbal or non-verbal) between one, or both, of the people in a conversation. These signals can indicate when someone is ready to transition from talking to listening, or when someone is willing or wants to take the floor from someone that is currently speaking. Attempts have been made to identify the variables that might contribute to turn-taking behaviour during natural interactions [14], and one of the most comprehensive theories was offered by Duncan and Fiske [2]. The authors conducted exploratory research, in a natural setting, where many observable behaviours (e.g. head nods, smiles) were recorded and coded. Participants were asked to sit and talk to each other for 7 minutes and almost 50 different behaviours were recorded for analysis. This resulted in a very rich data set collected during a natural interaction. For example, head facing direction was coded as an analogue for a person’s gaze direction and it was found that people gaze more *frequently* when they are speaking (presumably to monitor for understanding), but that people gaze for *longer* when they are listening. This finding has been replicated by other researchers [15,16], and the general pattern is thought to be that individuals hold their gaze on their partners when listening (with few averted gazes), but the proportion of direct to averted gaze are more evenly distributed when individuals are speaking. That said, it is important to note that some researchers have found considerable variability in both how often speakers gaze at a listener (20–65% of the time) and how often listeners gaze at a speaker (30–80%) [1].

Aggregated methods of analyses have provided important insights into the role of gaze during interactions. For example, it has been shown that gazing at a conversation partner occurs more in cooperative (compared to competitive) situations [17], that averting gaze away from one's partner tends to occur under cases of high cognitive load [18], that gaze is utilized more when one seeks disambiguating information from a partner [19], and that gaze can be used to regulate social interactions [20]. Collectively this suggests that gaze is an important part of the repertoire of signals displayed by each member of the dyad during interactions. However, the data that supports this conclusion was aggregated across entire sessions and this is a less than ideal, albeit typical, method for analyzing interaction data as the specific dynamics of turn-taking are lost.

### Turn-taking behaviour

Attempts have been made to look at transitions in turn-taking, and this has been described in the form of a “turn system” [2], suggesting that speakers often signal “transition ready states” that indicate their desire to pass the turn to their interaction partner. These signals are a collection of observable behaviours (e.g., head and eye direction) [21], and there are reports of a positive correlation between the number of these signals and the smoothness of the turn transition from one person to the other [2]. Critically, however, the specific temporal nature of these transitions has not been assessed, though it was noted [2] that this would offer useful insights into understanding dyadic interactions.

While gazing-toward and gazing-away from a partner may be driven by different mechanisms and is potentially context dependent [17–19], there is good reason to hypothesize that both mechanisms are related to the way turn-taking is regulated during interactions. Gaze behaviour, both toward and away from a partner, correlate with speech and turn transitions [22], and can signal different intentions. For example, eye gaze aligns with turn transitions such that speakers tend to end speech utterances with a gaze at their partner [1], presumably to signal that the turn is ending and to provide the partner with an opportunity to take the floor. Speakers also tend to look away as they begin talking [1,2,23]. It has therefore been suggested that gaze fills both a monitoring and regulating role [24], and this is supported by the observation of gaze differences between speech endings that occur at the end of phrases and ones that occur due to mid utterance hesitations [1]. Kendon [1] found that speakers gaze at their partner as they are about to end their phrase, but during hesitations averted gaze is found instead. The implication is that speakers gaze towards their conversational partners as a way to signal to them that they are ready for a turn transition to occur, but during times when they are not ready to give up the floor (e.g. during hesitations) they avert gaze to indicate that they want to retain their role as speaker. That said, it is important to note that a conversation is a two-way street, and speakers are not solely responsible for turn-taking [25]. For example, listeners have been found to gesture more and make more head/gaze shifts prior to speaking, possibly as a way to request a turn shift [26].

Much of the work presented thus far represents the quantitative support for the initial theoretical models [1,2] of signaling in conversation. And some of these studies were conducted in settings that allowed conversations to flow naturally (e.g., [1,23,25]). However, most are limited by the fact that they employed aggregated methods of analysis. In light of the recent and growing interest in natural social attention, an area that deserves additional focus is the temporal relationship between gaze and speech. Indeed, it was noted in the earliest work that this could offer insights into how interactions unfold over time, as well as the precise function of social gaze [2].

### Gaze and non-linear methods

The aggregation method of analysis typically used in the study of social attention collapses across time to calculate an average (whether it be an average gaze duration, or average number of fixations etc.). This method is useful for highlighting broad patterns in social interactions, but one shortcoming is that it distills an entire time series into a single data point, which obscures any temporal dynamics that may exist. For more granularity in the analyses of interactions, we should turn to an analysis method that is sensitive to changes over time.

Non-linear analysis methods allow one to examine how variables interact with each other, without confining them to a single time point. The most recent works in the quantification of social interactions have employed Cross Recurrence Quantification Analysis (CRQA). This method allows one to determine the percentage of co-occurring events in two time series, sampled across all possible time points. For a more extensive discussion of non-linear methods and CRQA, please refer to [27,28].

In the earliest usage of CRQA in studying interaction behaviour, speakers and listeners were asked to look at an image on a computer screen. Both participants were eye tracked as the speaker talked about the image, and a high co-occurrence between the gaze of the speakers and listeners was found, with the listeners lagging behind speakers by about 2 seconds [29]. Emphasizing further the importance of this temporal analysis, stronger coupling between the gaze of both dyad members was discovered to lead to higher levels of listener comprehension. In a later study, the gaze of speakers and listeners was again found to be tightly coupled, especially if both participants shared the same knowledge base [30]. These advances exposed a new domain of analysis methods for studying social interactions, however, they have thus far been limited to interacting with a shared reference point (i.e., a computer screen). In order to study social attention in interactions, one would ideally study an interacting dyad in a face-to-face situation.

The prevailing models of social signaling [1,2] give a good indication of the broad picture of natural social interactions, providing insight into, for example, the difference in gaze duration between speaking and listening. However, as noted previously, these studies are limited by the fact that they often focus on only one member of the dyad, use head direction as rough analog for gaze direction, and have generally aggregated gaze information over the course of an interaction, which prevents temporal dynamics from being observed. The recent studies using non-linear methods have highlighted the importance of timing and gaze information between speakers and listeners, but these methods have not been utilized in natural face-to-face settings.

Here we combine the two approaches to study the temporal nature of dyadic interactions in natural situations. Our novel methodology allows us to validate the findings from signaling models by using more accurate eye tracking technology to simultaneously assess gaze behaviour of both participants with a higher degree of precision. Furthermore, while others have studied temporal dynamics in computer based studies, we detail the temporal relationship between gaze and speech during a natural face-to-face interaction. In doing so we draw inspiration from a small handful of studies that have recorded the eye movements of multiple parties simultaneously during natural conversations (e.g., [22,31,32]) and which have helped to lay the groundwork for our present attempt, which primarily aims to provide a detailed description of the temporal dynamics within these interactions.

In summary, the present study examines the signaling approach originally described by Duncan and Fiske [2] and Kendon [1], using the latest social attention methodologies and analysis techniques. We concurrently eye track both members of a dyad during a natural face-to-face interaction to examine how gaze, talking, and turn-taking are related. We will use a non-linear method of cross-correlations to uncover the way in which gaze and speech are related in time, with the predictions that 1) prior to the end of speaking, speakers will signal the turn transition by gazing at their partner, and 2) a listener's transition to speaker begins with eye gaze averted. This is in support of currently existing models and data patterns, which suggest that gaze functions as a turn-taking signal and can guide the flow of conversation.

## Method

### Ethics Statement

Ethical approval was received through the University of British Columbia’s Behavioural Research Ethics Board and written informed consent was obtained from each participant prior to the start of the study.

### Participants

A total of 40 undergraduate students (mean age: 24.4 years, *SD* = 4.57, 16 male) were recruited from the University of British Columbia. Participants took part in one dyad each. 20 dyads were created from the 40 participants, and each participant was paired with the person who signed up for the same experimental session. Two participants (from the same pair) were completely removed from the analysis due to a computer related issue that prevented data recording. One participant was also missing gaze data in 20 Questions due to another computer issue. Therefore, our effective sample size was 38 participants in the majority of cases, and 37 in cases where 20 Questions or gaze information was required for the analysis. All participants received monetary reimbursement ($15) for their time. A post-test questionnaire was administered to determine participants’ attitudes towards various aspects of the task as well as collecting demographic information.

### Apparatus

**Fig 1:**
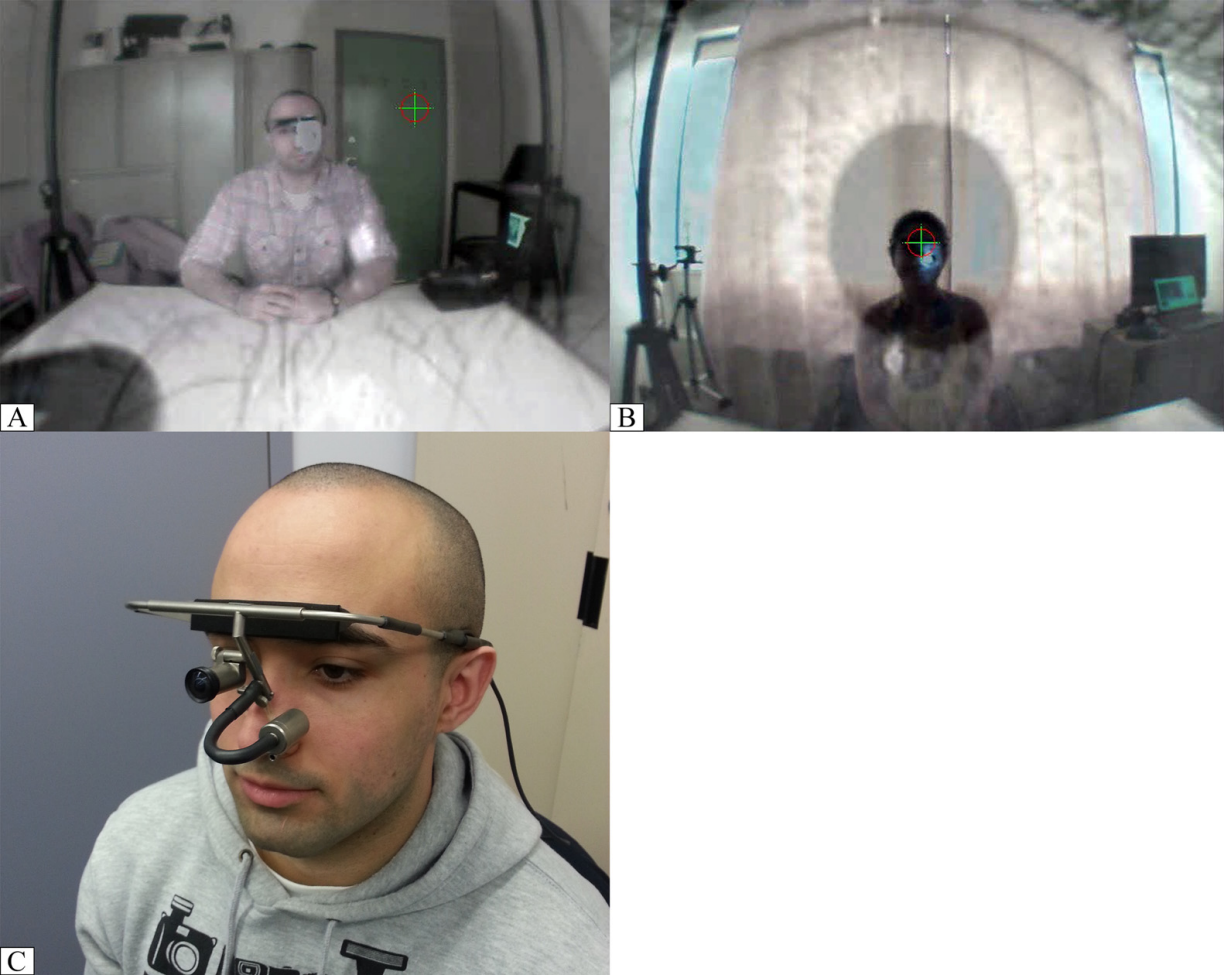
Dikablis eye tracker and associated video streams. Each Dikablis has a forward facing camera capturing a live video stream of the other participant, and the eye gaze of the wearer is overlaid on the video (A-B). Dikablis eye tracker worn by a participant (C). The individuals in this manuscript have given written informed consent (as outlined in PLOS consent form) to publish these case details.

Two Dikablis mobile eye trackers (http://www.ergoneers.com) were used to track the gaze of both participants. The Dikablis has two cameras; one recording the wearer’s eye, and one facing forward to record the wearer’s field of vision. The video feed from each Dikablis streamed into its own Dell laptop running Dikablis recorder software, which recorded and stored the video information from the wearer at a rate of 25 frames per second (see Fig 1A and 1B for an example of these video streams). After the experiment, the video feeds were checked and corrected for calibration in Dikablis analysis software to ensure the accuracy of the eye tracking.

### Procedure

Participants were asked to sit at opposite sides of a table, after which two research assistants put the Dikablis on each participant and went through a calibration process to ensure eye tracking was accurate. Given that we were utilizing two eye trackers, it was vitally important to make sure the video streams from each Dikablis was synchronized. This was achieved with a clap of the hands within the field of view of both eye trackers after the calibration phase. During the video coding phase we lined up the clap in the two video streams to ensure they were in sync with each other.

We asked the participants to briefly introduce themselves to their partner to 1) get them used to wearing the eye trackers and 2) help them feel more comfortable with each other. They were provided with a list of games and a rule sheet for each game. Participants were instructed to look at the games and to decide between themselves which game they would like to play first. The available games were “Heads Up” and “20 Questions”. Once they had decided on a game, the research assistants set it up, gave a brief description of the game, and then left the room while the participants played. The reason for leaving the room was so that we could assess a true dyadic interaction, in a natural way, without the presence of a researcher potentially affecting the gaze behaviour of the participants. After the game had ended, the researchers came back into the room to calibrate the eye trackers again. At this point the participants played their next game. The order of the games was up to them to encourage discussion about what they should play next.

We chose games that offered a balance between participant freedom and experimental control. Participant roles are clearly defined in each of the games and this structure facilitated comparisons between different dyads. Although the setup is not as relaxed as a spontaneous interaction between two people, we offered participants complete control over their own actions within the structure of the games. For example, participants can take as much time as they need at any point in the interaction and are allowed to act in any way they see fit. We further increased participants’ level of control by giving them a choice over the order of the games.

#### Heads Up

Our variation of Heads Up blends the original game with another guessing game: Taboo. In this game, a series of common words are written on individual flashcards, with one only word per card. The stack of cards is placed face down on the table. One participant is the explainer, who draws a card and attempts to describe the word on it without saying the word itself. The other participant is the guesser and their role is to try to guess that secret word as quickly as possible. This phase has a time limit of 3 minutes. Once the time is up, the participants switch roles and play for another 3 minutes. The goal of this game is to get through as many of the words as possible within the time limit. In the original Heads Up game, it is the guesser who turns over and holds up the card containing the word to be guessed, whereas in our version it is the explainer who turns over the card. While in Taboo the drawn card contains a list of prohibited words, our version only contains one prohibited word per card. In this game, the explainer does most of the talking and also has knowledge of the answer, whereas the guesser does very little talking and has no knowledge of the correct answer.

#### 20 Questions

In this game, one participant thinks of an object, place, or a person and the other participant is the guesser. The guesser has a maximum of 20 yes/no questions that they can ask to try to figure out what the item is. Once a total of 20 Questions have been asked, or the item has been guessed, the participants switch roles and a new item has to be guessed. The goal is to try to guess the word with as few questions as possible. In this game, the guesser does most of the talking and has no knowledge of the answer, whereas the participant who chose the item does very little talking but has knowledge of the correct answer.

### Video Coding

Once all the video data had been recorded and corrected for calibration, the research assistants went through the process of manually coding the video data. Adobe Premiere Pro CS5 (http://www.adobe.com) was used for the manual coding. We coded the start and end time of every individual gaze the participant made to their partner throughout the duration of the game—defined as when the eye gaze cursor enters the face region of the other participant, and the time when it left the same region. We also coded when each person started, and stopped, talking in each game. The combination of these data types allowed us to look at the relationship between gaze and talking in the two games. The full set of start/stop times can be found in S1 Dataset.

## Results

**Fig 2:**
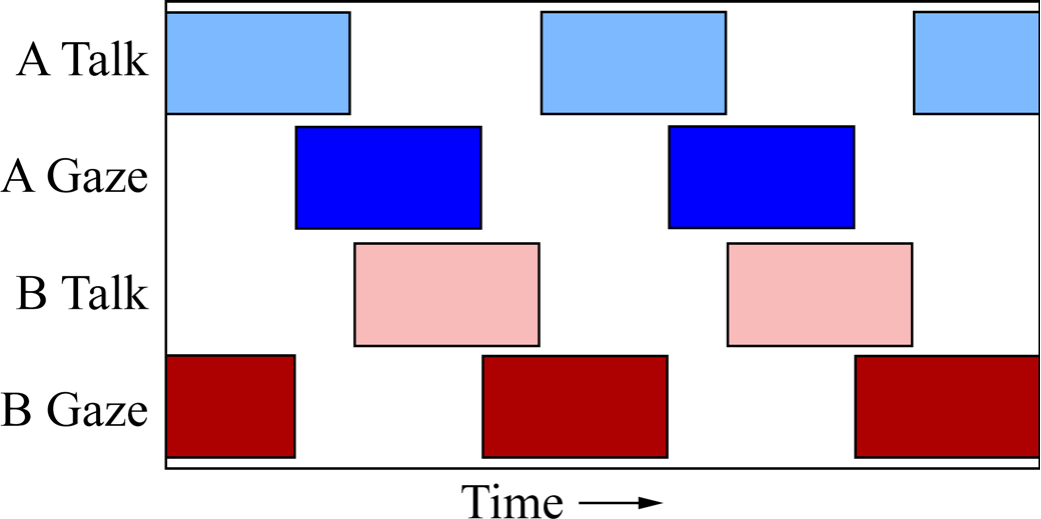
Idealized time series showing data pattern as predicted by earlier models of social interaction. Firstly, participants end their talking turn with direct gaze at their partner. This is represented by the onset of direct gaze (dark blue) occurring prior to the end of talking (light blue). Secondly, participants will be averting their gaze prior to speaking. This is shown by participant A’s talking turns (light blue) beginning with no direct gaze (dark blue). Both of these predictions are also depicted in participant B’s talking (light red) and gazing (dark red) series.

The turn-taking models presented earlier lead us to two predictions: 1) prior to the end of speaking, subjects will signal the turn transition by gazing at their partner and 2) prior to the beginning of a speaking turn, the subject will be averting their gaze. An idealized form of these predictions is shown in Fig 2. We began by describing general patterns in the relationship between gaze and speech, with an emphasis on identifying similarities and differences between the two games. The goal of the descriptive analysis is to provide an example of what gaze and speech behaviour look like in these games, and the observations are then supported by statistical analysis to quantify the patterns across dyads.

### Descriptive analysis

**Fig 3:**
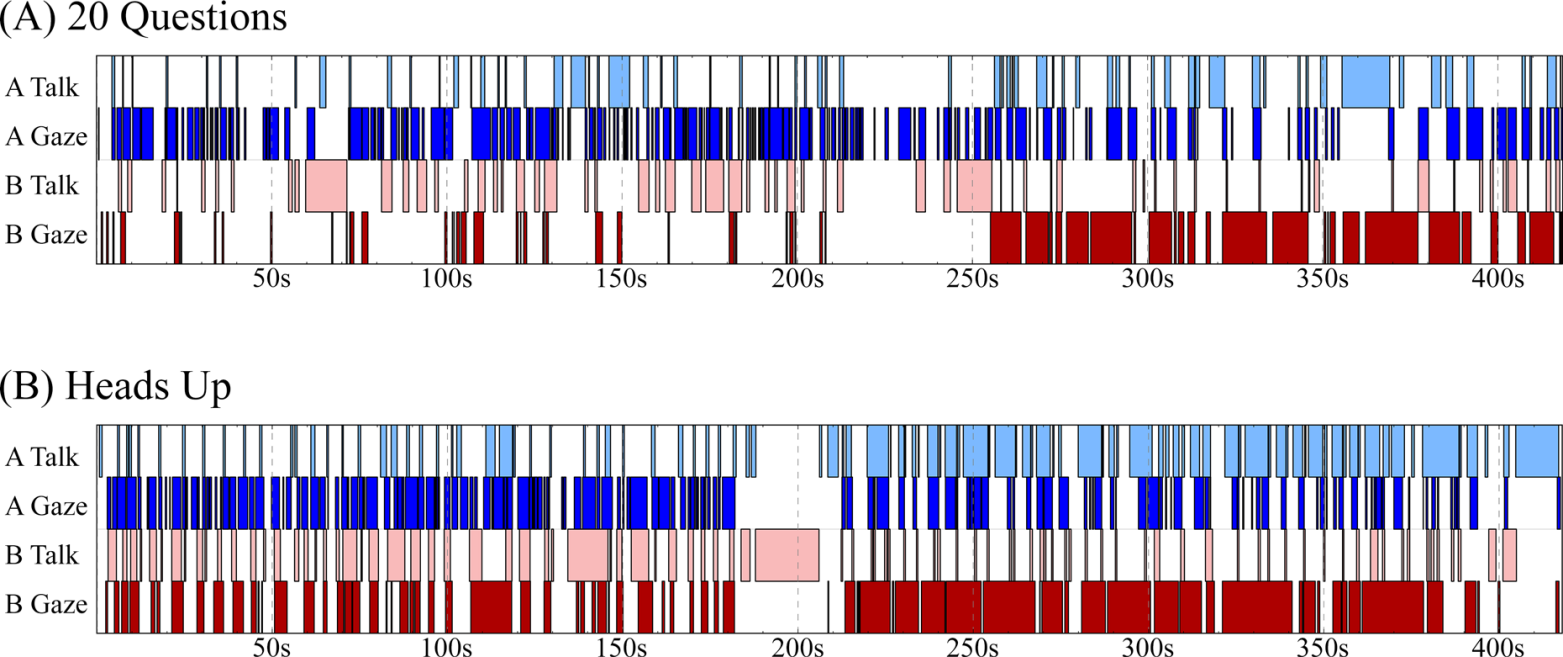
Time series of events in a single pair for 20 Questions (A) and Heads Up (B). Each series represents the duration of the entire game for a single dyad with the on/offset of all gaze and talking events from both participants. Within each series, the rows represent (in order from top to bottom): person A talk events, person A gaze events, person B talk events, person B gaze events. Of note is the density of each of these events at various points of the game. There is an inverse relationship between gaze and talking, such that when a participant has a higher density of talking events, there is an lower density of gazing events, and vice versa.

Taking an example of the two games from the perspective of a single pair, we can see from a cursory glance that there are some differences in gaze/talking behaviour between 20 Questions (Fig 3A) and Heads Up (Fig 3B).

As Fig 3 shows, the relationship between gaze and speech over the course of an interaction is complex. For example, speech events appear to be more spaced out in 20 Questions than in Heads Up. We tested this by comparing the average time between speakers in both games. For each game, we took each pair and calculated the average time between one person stopping talking and the other person starting. This resulted in an average value for each of the 19 pairs in the experiment, for which we found a significant difference between the two games (paired t-test across dyads, *t*(18) = 3.94, *p* = .001). The analysis suggests that speaking events are more spaced out in 20 Questions (2208ms) than in Heads Up (1430ms). Gaze behaviour was also different across the two games for this pair. As previously mentioned, one participant was missing gaze data for 20 Questions, so for the remaining 18 pairs we calculated the average time between one person stopping gazing and the other person starting. We found a significant difference between the two games, *t*(17) = 2.41, *p* < .05, suggesting that gazing events are more spaced out in Heads Up (2272ms) than in 20 Questions (1574ms).

One similarity between the two games is the inverse relationship between gaze and speech. As we will show below in the cross-correlational analysis, the general pattern observed is that when we are talking to someone we tend to be averting our gaze, and when we are listening we tend to gaze directly at the speaker. Fig 3B shows this pattern quite clearly. In the first half of this excerpt, person B is doing most of the talking (they are, in this case, the “explainer”), as they have a higher density of talk events (light red) compared to person A (light blue). Being in a talking role, person B spends less time gazing and they have a lower density of gaze events (dark red) compared to person A (dark blue). In the second half, the roles reverse and person A takes over as the main speaker with a higher density of talking events (light blue) compared to person B (light red). Gaze behaviour is sensitive to this change, and person B (dark red) now spends more time looking at person A (dark blue) than vice versa.

**Fig 4:**
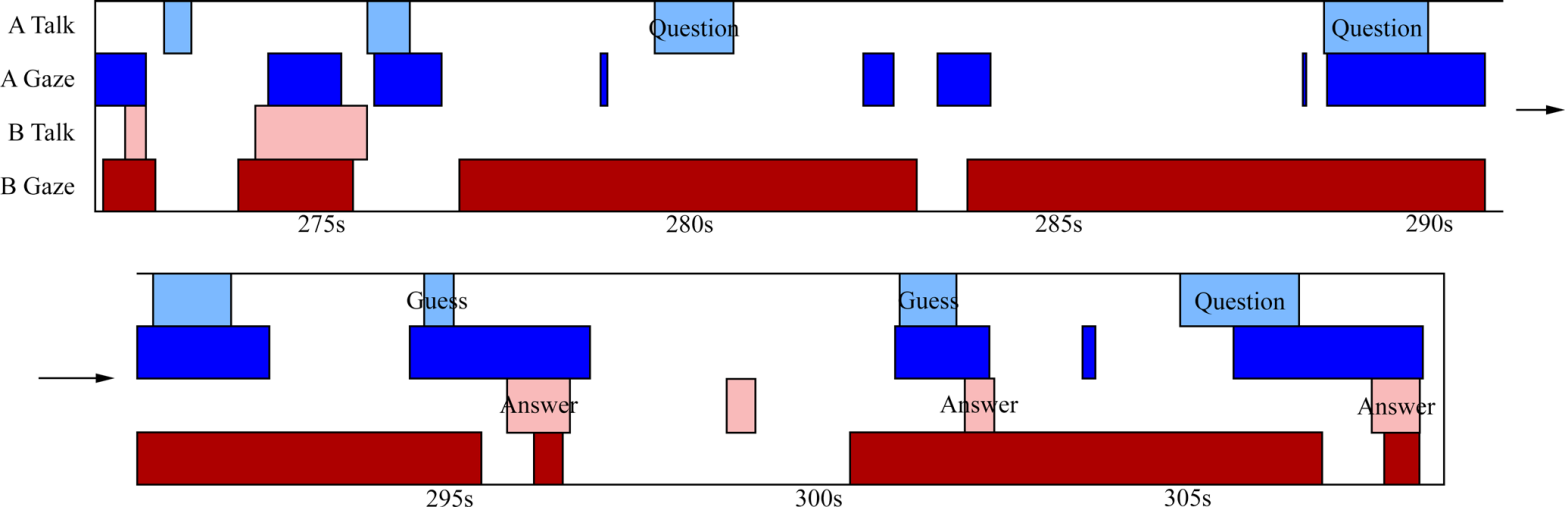
Segment of the 20 Questions game for a single pair. A short 20 Questions segment for a single pair of participants. Bottom half of the figure is a continuation of the top. Rows follow the same ordering as in Fig 3. Key talking events have been annotated. In this segment, person B thought of an object while person A is guessing/asking questions about it. As with Fig 3, an inverse relationship is seen between density of gaze and talking events. Furthermore, talking events are generally preceded by the same participant averting their gaze.

**Fig 5:**
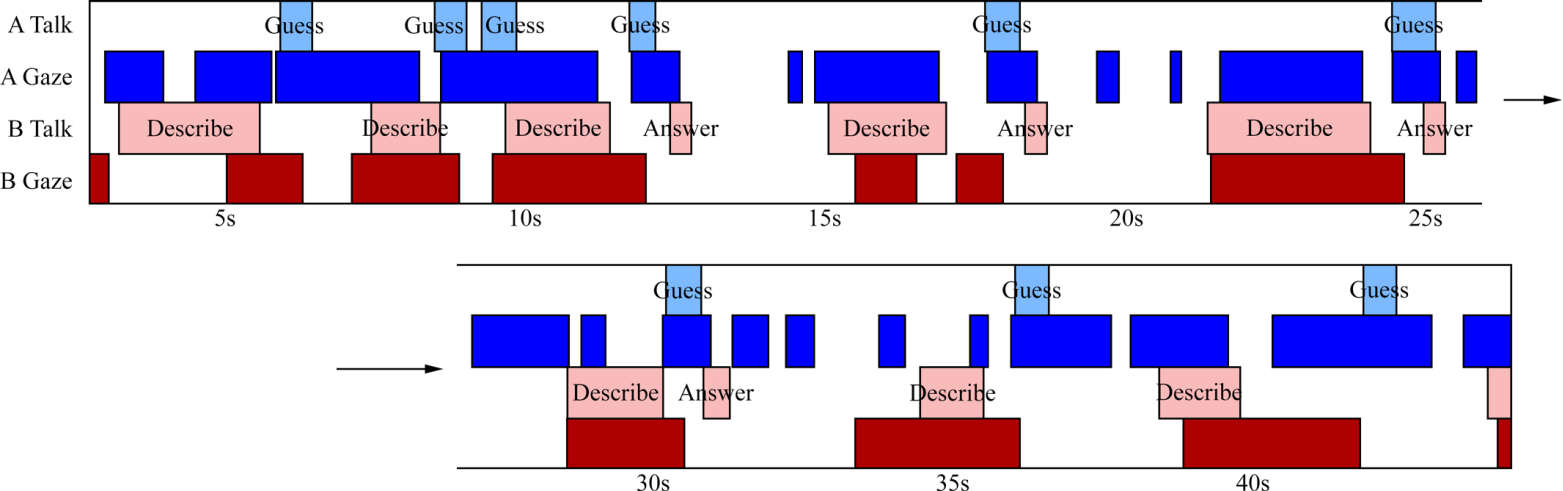
Segment of the Heads Up game for a single pair. A short Heads Up segment for a single pair of participants. Bottom half of the figure is a continuation of the top. Rows follow the same ordering as in Fig 3. Key talking events have been annotated. In this segment, person B is describing the object while person A is guessing its identity. As with Fig 3, an inverse relationship is seen between density of gaze and talking events. This is especially evident for person A. Furthermore, speech turns typically begin with averted gaze, and end with direct gaze.

Next, we took a closer look at the relationship between gaze and talking, in both games, to highlight some additional patterns of behaviour. Fig 4 shows the gaze and speaking events during a small segment of an interaction in the 20 Questions game, for the same dyad as depicted in Fig 3. For both participants, talking events are often preceded by averted gaze—talking begins while looking away from the other person (demonstrated at 276s, 288s, and 305s for person A). There is also a tendency to start talking only after a direct gaze has been received from the listener. Fig 5 shows a short segment from the Heads Up game for the same dyad. As with 20 Questions, talking events are generally preceded by an averted gaze by the speaker (e.g., 15s, 21s, 28s, and 38s for person B), and they tend to start talking when the other person is already gazing at them. Furthermore, speech turns tend to end with direct gaze at the partner (e.g., 5s, 30s, and 39s for person B).

### Cross-correlation analysis

Thus far we have employed a descriptive approach to assess the relationship between gaze and talking of the two participants. A useful way to quantify the differences and similarities between these event types is to employ a cross-correlational analysis; a correlation based method typically used in the fields of digital signal processing and neuroscience (e.g., [33,34]).

Each time series exists as a binary and discrete series measured across time. Each point in time, or specifically each video frame in this case, can be assigned either a 0 or 1 for the event type it is measuring. In a gaze series a 0 would indicate averted gaze while a 1 would indicate direct gaze at the other participant. In a speech time series, moments of speech and silence can be represented by 1s and 0s respectively. The time series can then be compared both within and between participants, for multiple variable types, using a cross-correlation. We assess the magnitude of similarity between the two series using a general correlational method. The correlation between two aligned sequences is known as the 0 lag correlation. One series is then shifted by 1 frame, relative to the other series, and the correlation calculated again. We do this for all possible time shifts (both forward and backward in time), resulting in a correlation value for each time lag. The maximum correlation value is then normalized (between 0–1) for ease of comparison between series of differing lengths, and then used for analysis. All analyses used the maximum cross-correlation value, unless otherwise stated. Calculating the cross-correlation between two series will give us 1) the maximum amount of similarity between the two series and 2) the lag of this similarity value, which is a measure of how many seconds/frames the series must be shifted by (in time) in order to achieve that maximum value. For participant-level analyses we calculated the cross-correlation for each person and then averaged across all participants. For dyad-level analyses, a cross-correlation was calculated for the dyad, and then averaged across all dyads.

**Fig 6:**
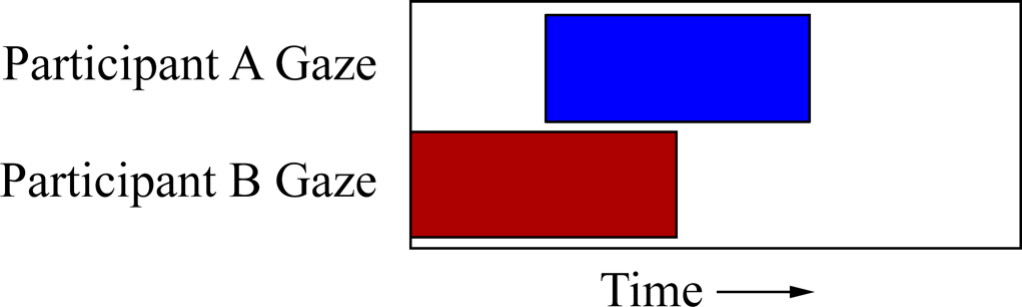
Simplified example demonstrating the cross-correlation technique. An example of how a cross-correlation can be used to measure the temporal relationship between partners’ gaze, where a solid block represents the onset of a gaze event and white space represents averted gaze. Here, the gaze of participant B precedes that of participant A (see text for more detail).

To demonstrate this technique, we can use a simplified example. Let us imagine we want to compare the similarity between the gaze series of two participants. Participant A’s binary series could be [001111000], which represents averted gaze followed by 4 frames (160ms at 25fps) of direct gaze and ending with gaze averted again. Participant B’s data might be [111100000], indicating direct gaze followed by averted gaze. Both series are shown visually in Fig 6. A cross-correlation, with no lag, is calculated using element-wise multiplication of the 2 vectors and summing the result, which gives a value of 2 (0x1 + 0x1 + 1x1 + 1x1 + … + 0x0). To normalize, we divide it by the product of the 2 vector norms (2x2), which gives a normalized cross-correlation value of *r* = .50. Participant B’s series is then shifted by 1 frame to [011110000] and the normalized cross-correlation calculated again (*r* = .75). As the 2 series become more similar, the value of the cross-correlation gets larger. This process continues for every single possible time shift (both forward and backward), giving us a collection of correlation values and their associated time shift.

In terms of the cross-correlation between these two, we can see that both participants spend the same amount of time making direct gaze, but that participant A starts gazing 2 frames (80ms) later. The cross-correlation for these data gives a maximum correlation of 1.0, at a lag of +2 frames. In other words, the gaze behaviour of both participants is highly similar (they both spend 4 frames gazing at the other person), but participant A has a +2 frame (80ms) lag in their data suggesting that their gaze follows that of participant B. The same technique can be utilized to test the hypotheses generated from the earlier models.

In this study, we used this cross-correlational approach to compare both gaze and talking data, across the entire duration of a game. Python was used to calculate the cross-correlation and lag values, and the numbers were also verified using MATLAB. In practice, because we were interested in turn-taking transitions rather than larger scale rhythmic patterns, we constrained the range of lags to two seconds before or after an exact match between signals. This was a reasonable figure given the timings that we observed, and is within the range of limits used by other researchers [1,23,29,35]. For analyses at the dyad-level, correlations and maximum lags (in ms) were computed for each dyad, separately for each game, and then averaged across dyads. For participant-level analyses, correlations and maximum lags were computed for each individual participant, for each game, before being averaged across all participants.

#### Is there similarity in gaze / talking between dyad members?

We began by assessing the similarity between the gaze signals of the dyad members, using a traditional Pearson family correlation as we were not concerned with time shifts between the signals. Point-biserial and phi correlations are alternative analyses, but are mathematically equivalent to the Pearson product-moment correlation. These correlations were computed for each pair (resulting in 19 correlation values, and 18 when the analysis called for 20 Questions gaze data) using the entire game duration and a mean was then calculated by averaging across all dyads.

In 20 Questions, a negligible mean correlation was found between the 2 gaze signals, *r* = -.05. This value was tested against a zero correlation and found to be non-significant, *t*(17) = 1.77, *p* = .096. In Heads Up, a significant negative correlation was found, *r* = -.18, *t*(18) = 14.61, *p* < .0001. We compared the correlations between the 2 games and found them to be significantly different, *t*(17) = 3.35, *p* = .004.

These results suggest that there is little consistency in the gaze of the dyad members when playing 20 Questions. However, the significant negative relationship found in Heads Up indicates a back-and-forth in gaze behaviour—as one person gazes, there is a tendency for their partner to be looking away. These gaze patterns are consistent with the nature of the games. Back-and-forth interaction is strongly encouraged in Heads Up, where the game unfolds in a rapid question-answer format due to the imposed time limit. On the other hand, 20 Questions is a slower game where question-answer interactions have a more relaxed structure and there are longer periods of silence.

Next, we looked at the similarity in talking signals of dyad members. No reliable correlation was found in 20 Questions, *r* = .08, *t*(18) = 1.69, *p* = .11, while a significant negative relationship was found in Heads Up, *r* = -.26, *t*(18) = 14.08, *p* < .0001. As with the gaze signals, comparing the talking correlation values between the 2 games yielded a significant difference, *t*(18) = 7.84, *p* < .0001. These results support the notion of Heads Up being a more interactive game, where both the gazing and talking signals of the pair members suggest turn-taking behaviour (i.e. when one person gazes, the other averts their gaze. When one person talks, their partner is silent).

Assessing the gaze and talking signals independently provides some useful insight into how the interactions unfold. However, in order to test the predictions derived from earlier theoretical models we turn to cross-correlational analysis in order to assess the temporal relationships between these variables.

#### Do speakers gaze to signal end-of-turn transitions?

**Fig 7:**
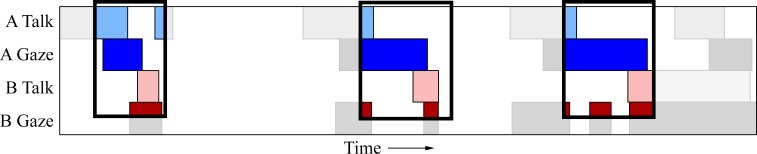
Excerpt from a Heads Up game showing gaze being used to signal an end-of-turn transition. The boxes highlight regions from the excerpt where the data pattern is particularly evident. Participant A gazes (dark blue) at their partner (B) prior to the partner beginning their speech turn (light red). Additionally, participant A tends to end their speech turn (light blue) by gazing at their partner (dark blue). The pattern suggests that direct gaze is used to end a current speech turn and also to signal a turn transition.

To test the hypothesis that speakers use gaze to signal the end of their turn we computed the maximum cross-correlation between a participant’s gaze and their *partner’s* talking. These analyses were conducted at the participant level, where each *participant* has a corresponding correlation value. This is in contrast to earlier correlation analyses, which were conducted at the dyad level, where each *dyad* generated a single correlation value. Participant level analysis resulted in 38 correlation values (37 when 20 Questions gaze data was required). In 20 Questions we found a high mean cross-correlation (across all dyads) between the two variables, *r* = .47, and the average lag at which this correlation occurred showed partner talking tends to lag behind participant gazing by 423ms (*SD* = 388ms). A t-test across participants showed a significant departure from a zero correlation, *t*(36) = 35.87, *p* < .0001. The same finding is seen in Heads Up, *r* = .53, *t*(37) = 24.83, *p* < .0001. As in the 20 Questions game, talking lags behind the other persons gazing, by 432ms on average (*SD* = 557ms). An example of this, from a single dyad, can be found in Fig 7. The boxes highlight sections where this pattern is particularly evident. In each box, we can clearly see that person B (light red) does not begin talking until person A (dark blue) gazes at them. Furthermore, person A’s speech turns are ended with direct gaze at their partner.

A significant difference was found between the cross-correlation values of the 2 games, *t*(36) = 2.79, *p* = .008. This suggests that, despite there being high similarity between a participant’s gaze and their partner’s speech in both games, the correlation is higher in the Heads Up game, presumably because the game is designed to be more interactive and there are fewer moments of silence. However, the lag values in the two games were remarkably consistent (20 Questions: 423ms, Heads Up: 432ms), and there was no significant difference, *t*(36) = 0.08, *p* = .94.

**Fig 8:**
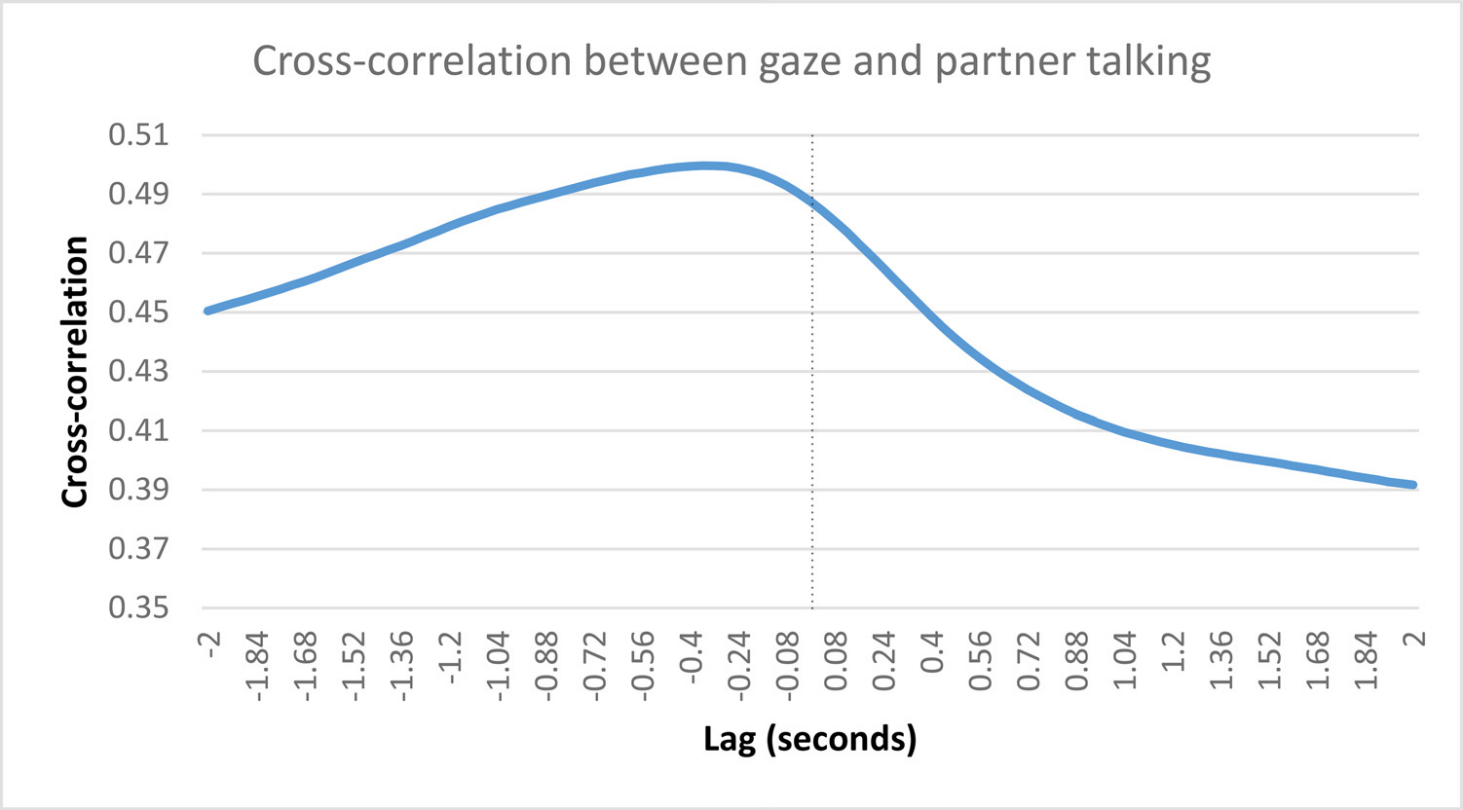
Cross-correlation values (participant gazing and partner talking) at each lag. Cross-correlation between participant gazing and partner talking computed at each lag value. The highest cross-correlation values occur at negative lags, when partner talking lags behind participant gazing.

We collapsed across both games and averaged every participant’s correlation, at each lag value, to show the how the cross-correlation unfolds over time. This relationship can be seen in Fig 8. The participant who was missing 20 Questions gaze data was removed for this visualization. The figure clearly shows that the highest cross-correlation values occur at negative lags, which, in this case, occur when partner talking lags behind participant gazing.

The data support the suggestion of earlier interaction models, which state that speakers end their turn with a gaze toward their partner, after which the partner begins their speaking turn in the interaction (e.g., [1]). Here, we have shown that a gaze toward a participant's partner occurs prior to them beginning to speak. Partner speech typically begins about 400ms after gaze onset for both games. This finding supports the general pattern observed in the descriptive analysis section. Fig 3 highlighted an inverse relationship between gaze and speech, such that moments of speech are related to averted gaze, while silence is associated with direct gaze. We confirmed this finding using a cross-correlational technique, where we show that direct gaze is associated with the end of a speech turn. That we observed this general behavioural pattern during specific moments of turn-transitioning is strongly supportive of the communicative function of gaze during social interactions.

#### Do speakers avert gaze at the beginning of their turn?

While direct gaze can signal end-of-turn transitions, it has also been suggested that averted gaze can indicate a desire to retain the current turn in the interaction. For example, speakers tend to avert their gaze when their speaking turn begins as a way to signal that they now have the floor [1,2].

**Fig 9:**
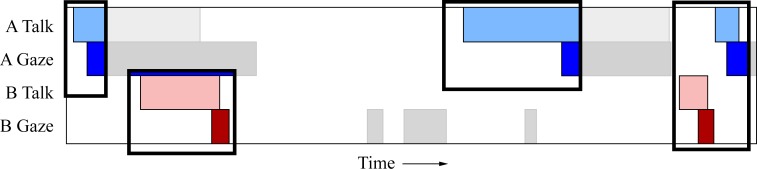
Excerpt from Heads Up showing gaze aversion at the beginning of a speaking turn. The boxes highlight regions from the excerpt where the data pattern is particularly evident. Both participants A and B begin their speech turn with averted gaze. Once the speech turn has started, they then gaze towards the other person.

To test this hypothesis, we calculated the maximum cross-correlation between a speaker’s gaze and their *own* talking. In 20 Questions we found a strong correlation between the two variables, *r* = .37, *t*(36) = 21.37, *p* < .0001. The mean lag indicates that a person’s direct gaze lags behind their own talking by 780ms (*SD* = 923ms). The same pattern is found in Heads Up, *r* = .40, *t*(37) = 18.78, *p* < .0001. Again, lag indicates that a person’s gaze follows their own talking by 736ms (*SD* = 724ms). An example of this, from a single dyad, can be found in Fig 9. The boxes highlight sections of the interaction where this behaviour is exhibited. Person A’s talking events (light blue) are preceded by averted gaze, and it is not until they have started to talk that they gaze at their partner (dark blue). The same pattern can be observed for Person B’s talk events (light red) and gaze events (dark red).

No significant difference was found in the cross-correlation value between the two games, *t*(36) = 1.11, *p* = .28. Nor was there a difference in the lag values (20 Questions: 780ms, Heads Up: 736ms), *t*(36) = 0.29, *p* = .77. This suggests that despite the two games having different rules, styles of play and requiring different social dynamics, gaze and talking behaviour at the beginning of a turn remains fairly consistent for each participant.

**Fig 10:**
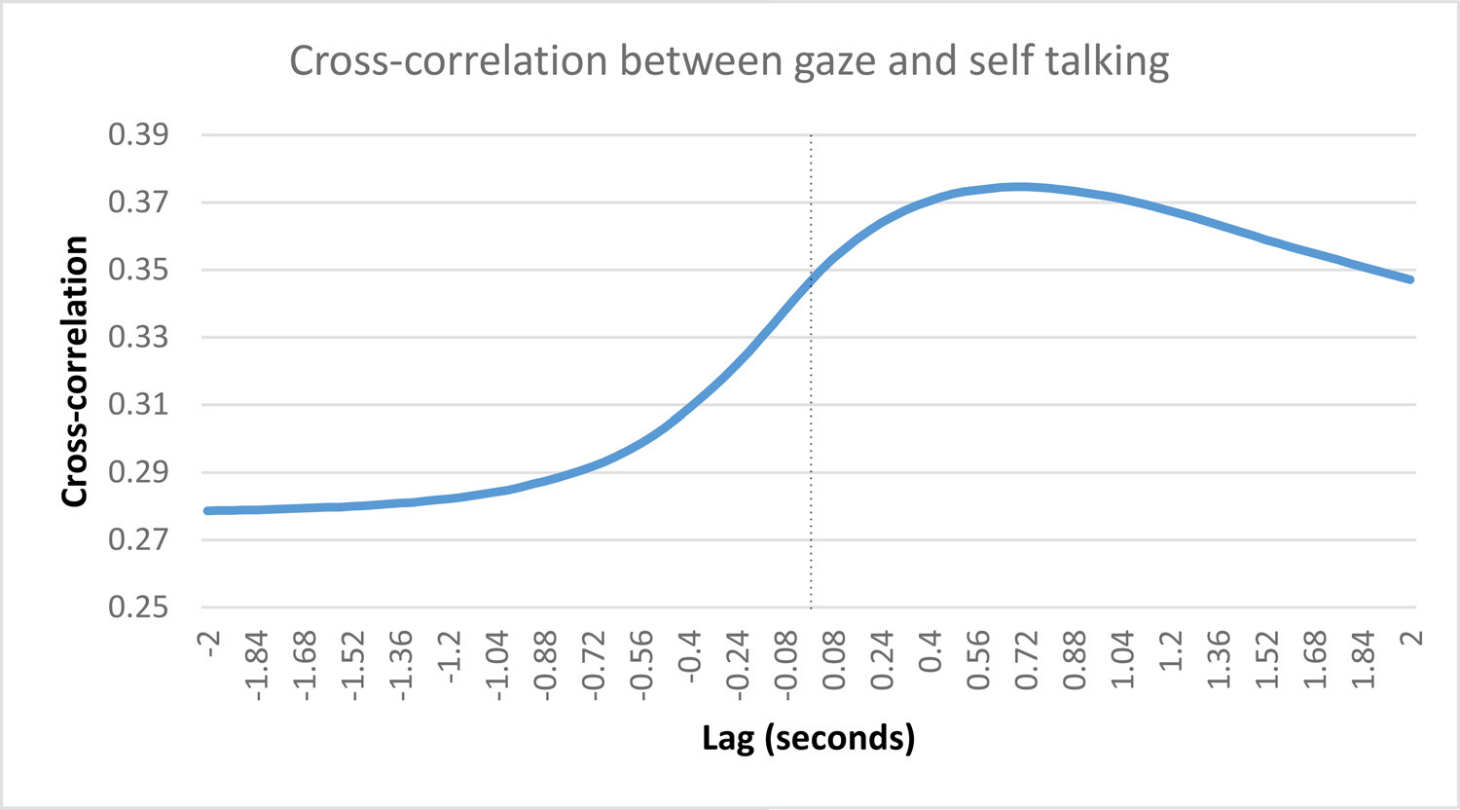
Cross-correlation values (participant gazing and own talking) at each lag. Cross-correlation between participant gazing and their own talking computed at each lag value. The highest cross-correlation values occur at positive lags, when participant gazing lags behind their own talking.

We collapsed across both games and averaged every participant’s correlation, at each lag value, to show the how the cross-correlation unfolds over time. This relationship can be seen in Fig 10. The participant who was missing 20 Questions gaze data was removed for this visualization. The figure clearly shows that the highest cross-correlation values occur at positive lags, which, in this case, occur when gazing lags behind talking.

## Discussion

The present study examined the signaling approach originally described by Duncan and Fiske [2] and Kendon [1]. We focused primarily on these models because they offered the most comprehensive analysis of signaling in social interactions, and serve as the backbone to many of the aggregated analyses of gaze behaviour that followed. These foundational studies reported that speakers tend to look away from their partner as they begin talking, and look back when they are about to finish their turn in the conversation. These observations suggest that eye gaze can be used to control the flow of conversation: we look away from our partner to signal that we have the floor and that it is our turn to talk, but when we are ready to hand control back to our partner we look back at them to signal them to begin their turn. Although these observations were reported for natural conversations, they lack both sensitivity to temporal dynamics of conversations and the resolution offered by eye tracking techniques. On the other hand, modern research on social attention employs accurate eye tracking methodologies, but has not studied attention in a real time dyadic interaction. The present study aimed to expand on these findings by employing modern eye tracking and analysis techniques, in a natural setting, to test previously observed patterns of signaling in social attention.

Using a cross-correlational approach we were able to compare the temporal alignment between direct gaze and speaking. There were two particularly noticeable patterns. Firstly, we found that speakers end their turn with direct gaze at their partner, after which a turn transition occurs and partner speech begins roughly 400ms later. This suggests that a speaker's gaze towards one's listening partner can signal a switch in roles. This pattern was found consistently across the two games, despite quite different structures in terms of the interaction. Although changes in gaze could come about for several reasons (e.g., one might look to gauge a partner’s reaction to an utterance), classic and recent research supports the interpretation that gaze is communicative [2,36]. Specifically, the systematic link between gaze and speaking, across members in a dyad, indicates that direct gaze is being used as a signal for turn-taking in a natural context.

In Foulsham et al. [11], observers watching video clips of a conversation tended to look at people in the clips slightly before these people started speaking (about 150ms, on average). In the current, face-to-face task, the average lag between looking at someone and them starting to speak was longer (400ms). Of course, when watching a video there were no real people and the observer did not speak and could not communicate with those depicted. That gaze still preceded partner speech may suggest that this is a stereotyped pattern, even when no communication is possible, or that viewers were responding to cues of turn-taking. An intriguing possibility is that gaze may occur earlier in real life because the recipient (who is about to speak) must perceive and interpret this signal so that they can be sure the current turn is over. Such nuanced signaling can obviously only be studied by taking into account both participants in a real interaction.

Secondly, we found that speakers tend to begin their turn with averted gaze, and it is not until around 700ms after they start talking that they gaze at their partner. Beginning a turn with averted gaze could signal a desire to maintain the turn, letting the partner know that they have the floor. Once the current turn-taker has been established, gazing back at the partner likely serves a monitoring function to check for understanding [24]. These two findings are in line with what Duncan and Fiske [2] and Kendon [1] observed originally during their studies of natural interactions. These findings are present in both games, which suggests that the relationship between eye gaze and talking remains consistent despite the changes in the structure of the interaction as imposed by the game type.

In a recent study by Freeth et al. [6], participants answering an interviewer’s questions tended to avert their gaze while speaking. This was a robust effect that was also found when a videotape of the interviewer asking the same questions was played to new participants being eye tracked. This suggests that, even if the function of averting gaze is communicative, it may be an overlearned response that is also elicited when communication is not possible. However, the Freeth et al. study did not look at natural patterns of conversation, and neither did it investigate the timing of gaze shifts in detail. The present data show that speakers begin talking with their gaze averted from their partner, but they later shift back to direct gaze.

We have argued that using a cross-correlational analysis provides novel temporal insights into the relationship between gaze and speech signals. A particularly interesting finding is that though gaze of the two members of the dyad are (anti) correlated in Heads Up, there is no such relationship in 20 Questions. The same is found when correlating the talking signals of the pair members. This underlines the difference in structure between the two tasks. Because there are longer and more spontaneous pauses in 20 Questions, there are often moments when neither party is speaking or gazing. Heads Up is a more conversational game, where rapid back-and-forth interaction is encouraged. Thus, a negative relationship emerges as when one person is speaking, the other is silent. Clearly, the direct relationship between gaze and speech is sensitive to task differences. That we found consistent results across tasks looking at the *interaction* between gaze and speaking makes this a promising pattern to investigate in future studies.

### Limitations and future directions

Both of our tasks were structured and had clearly defined participant roles. We chose structured tasks for two main reasons. Firstly, even naturally occurring interactions have defined roles. For example, when two friends are having a conversation one will typically be talking while the other listens, or when a student asks a question in class the professor will adopt a listening role before transitioning into a talking role. Real life social interactions naturally contain roles, and we made the roles explicit in our games. Secondly, explicitly defining roles is useful from an experimental control perspective as it allowed us to compare behaviours between dyads. Clearly there is value in studying interactions without explicitly imposing roles on participants and this will be a useful extension to the current study. Given that people naturally fall into roles during interactions we expect our results to generalize to situations where the interaction structure is more relaxed.

It is possible that initial gaze aversion may reflect the effect of cognitive load. It has been found that people tend to avert their gaze under cases of high cognitive load [18]. Our games are naturally high in cognitive load as participants were required to think of the best way to describe a particular object, or the best question to ask to discern an object’s identity. Upon taking the floor, it may be that cognitive load increases while one tries to think of what to say, or how to say it, which then leads to averted gaze. Future extensions could address this concern by manipulating cognitive load by varying task difficulty (e.g., using more uncommon words during Heads Up), or by using different situations ranging from free conversations to more constrained scenarios.

A unique feature of our study is the use of dual eye-tracking in real time. However, this comes with the natural limitation of possible distraction from a tracker being placed in front of each participant’s face. We have no evidence to suggest that this would impact the results, especially given that our data are in line with previous experiments on dyadic interaction that did not use eye trackers [1,2]. One might also wonder if the relatively slow video capture rate of our eye trackers (25 frames per second) adversely impacted our findings, for instance, resulting in our missing eye movements. As our interest was in *eye fixations*, that is, where the eye is positioned when it is not in motion, and not in the high velocity rapid *eye movements* called saccades, which would require a higher sampling rate to capture, we do not expect sampling rate to negatively impact our results. That said, it is an open question what effect frame rate has on gaze analysis.

Much of the recent research investigating attention to others has involved measuring gaze cueing effects, whereby participants attend to locations on a computer screen that are the target of a computerized gazing face [7–9]. However, real world studies on gaze following have sometimes found precisely the opposite pattern of results, with people often tending to look away from the target location of another's gaze [10]. This is partially supported with our 20 Questions data, where participants typically displayed opposing gaze behaviour, i.e., when one person gazes directly at their partner the other person averts their gaze. That said, the link between gaze behaviour in the present study and gaze cueing may be tangential at best, because as we have shown here, in real life dyadic interactions initiating and averting direct gaze tends to operate in service to communication between dyads, rather than with the aim of acquiring information at a gazed-at location as is the case with traditional gaze cueing.

This study is one of the first to employ an eye tracking technique where both members of a dyad are tracked simultaneously in a natural setting. Although others have made use of dual eye tracking (e.g., [32]), our study focuses on social settings and verbal interaction. Our move away from an artificial lab based study of social attention provides a glimpse into how attention operates in real world interactions, and the use of a cross-correlational approach allows the temporal dynamics of these interactions to be examined with greater resolution. Our results suggest that the eyes can be used to signal a turn transition using direct gaze, thus allowing the other person to take the floor, and also signal a desire to retain the current turn by averting gaze instead. These results, and our interaction paradigm, have the potential to reveal significant insights in future investigations into natural social attention.

## Supporting Information

S1 Dataset Gaze and talking start/stop times for all participants. (ZIP)
